# Supplementary material for: A yeast phenomic model for the influence of Warburg metabolism on genetic buffering of doxorubicin
Source: Cancer Metab. 2019 Oct 23;7:9. doi: 10.1186/s40170-019-0201-3 (PMC6806529; doi:10.1186/s40170-019-0201-3)
Supplement: Supplementary file 9 — Additional file 9. GO term-specific heatmaps for REMc/GTF-enriched clusters. GO term-specific heatmaps for significant GO process terms were generated as described in methods and Figs. 3 and 4. Any related child terms are presented in subsequent pages of the parent file name. GO terms with more than 100 children, with 2 or fewer genes annotated to the term, or a file size over 300KB are not shown. All heatmaps are generated with the same layout (see Figs. 3 and 4). [file 40170_2019_201_MOESM9_ESM.bz2 › Additional_File9_GOTermHeatmaps/Additional_File9_GOTermHeatmap/telomere_maintenance.pdf]

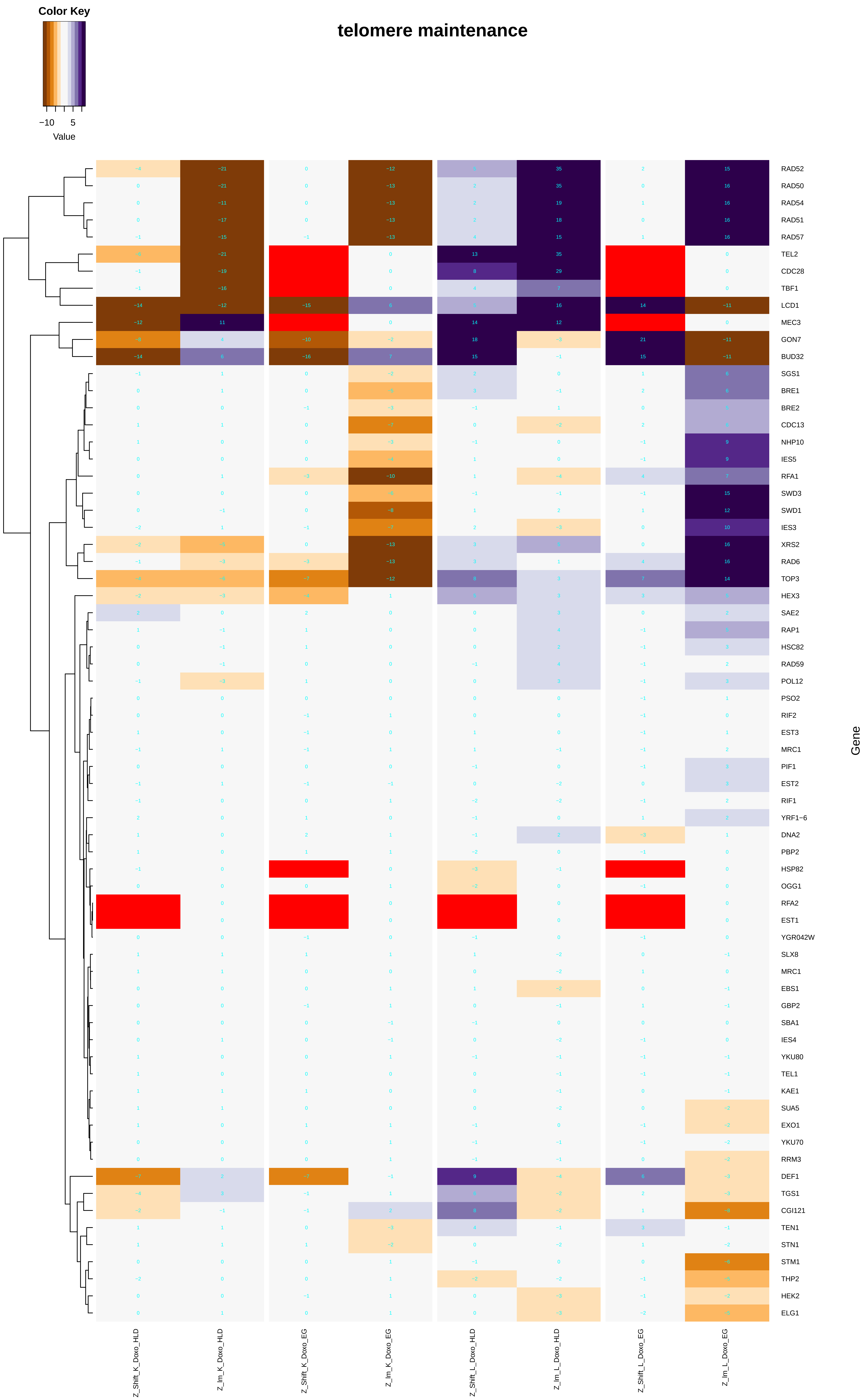

telomere maintenance
Color Key
−10
5
Value
Gene
RAD52
RAD50
RAD54
RAD51
RAD57
TEL2
CDC28
TBF1
LCD1
MEC3
GON7
BUD32
SGS1
BRE1
BRE2
CDC13
NHP10
IES5
RFA1
SWD3
SWD1
IES3
XRS2
RAD6
TOP3
HEX3
SAE2
RAP1
HSC82
RAD59
POL12
PSO2
RIF2
EST3
MRC1
PIF1
EST2
RIF1
YRF1−6
DNA2
PBP2
HSP82
OGG1
RFA2
EST1
YGR042W
SLX8
MRC1
EBS1
GBP2
SBA1
IES4
YKU80
TEL1
KAE1
SUA5
EXO1
YKU70
RRM3
DEF1
TGS1
CGI121
TEN1
STN1
STM1
THP2
HEK2
ELG1
Z_Shift_K_Doxo_HLD
Z_Im_K_Doxo_HLD
Z_Shift_K_Doxo_EG
Z_Im_K_Doxo_EG
Z_Shift_L_Doxo_HLD
Z_Im_L_Doxo_HLD
Z_Shift_L_Doxo_EG
Z_Im_L_Doxo_EG

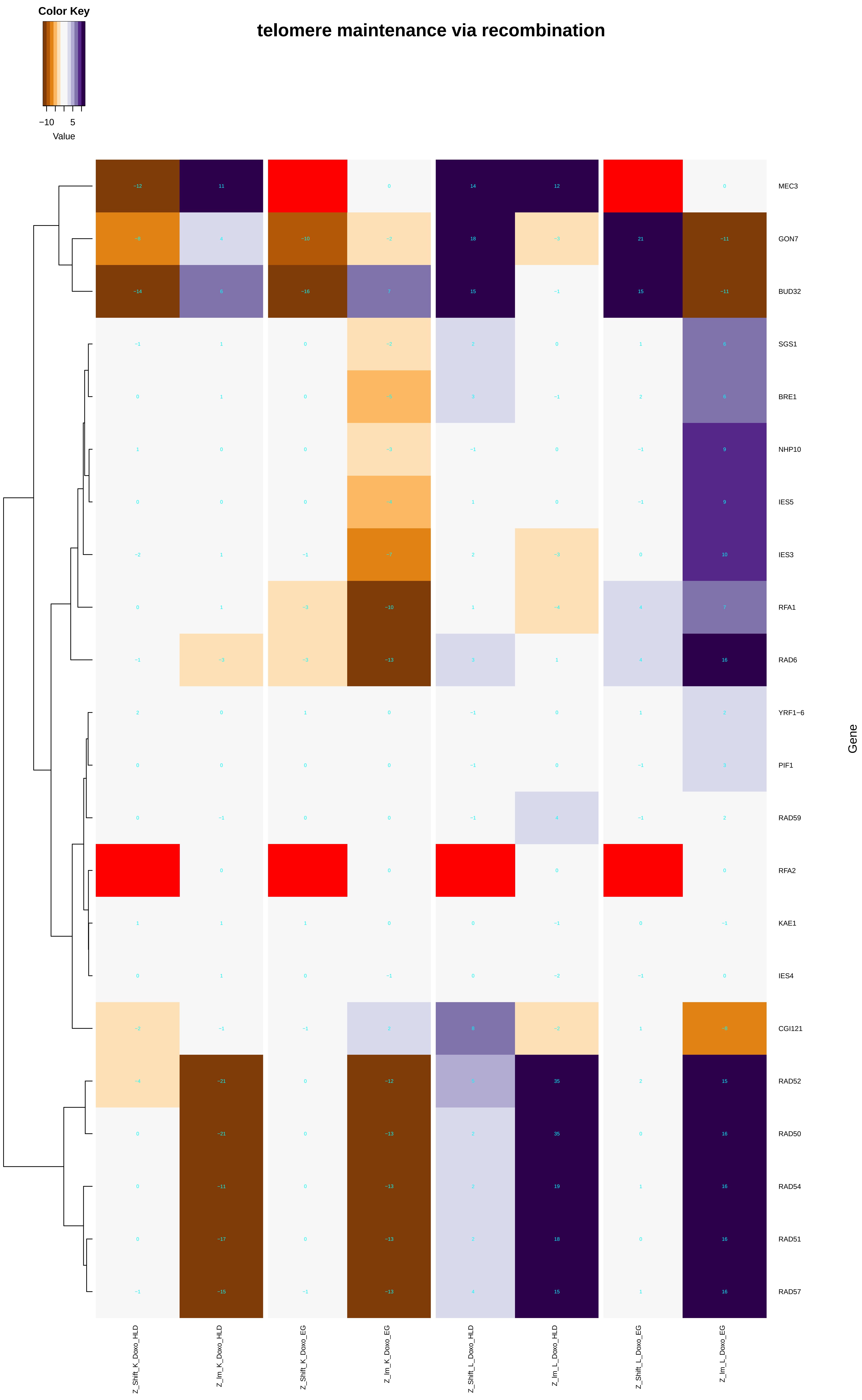

Color Key
−10
5
Value
telomere maintenance via recombination
MEC3
GON7
BUD32
SGS1
BRE1
NHP10
IES5
IES3
RFA1
RAD6
YRF1−6
PIF1
RAD59
RFA2
KAE1
IES4
CGI121
RAD52
RAD50
RAD54
RAD51
RAD57
Gene
Z_Shift_K_Doxo_HLD
Z_Im_K_Doxo_HLD
Z_Shift_K_Doxo_EG
Z_Im_K_Doxo_EG
Z_Shift_L_Doxo_HLD
Z_Im_L_Doxo_HLD
Z_Shift_L_Doxo_EG
Z_Im_L_Doxo_EG

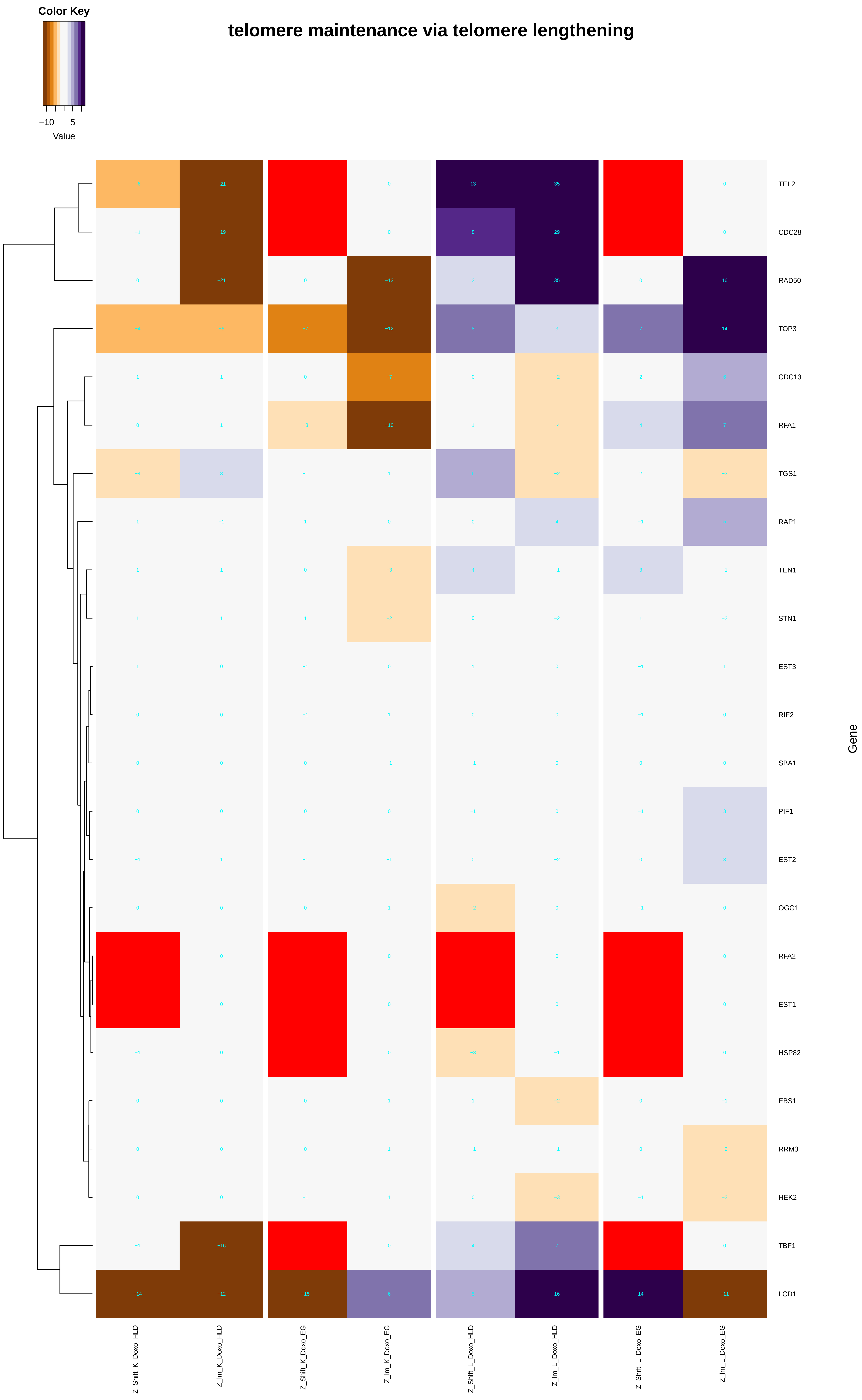

telomere maintenance via telomere lengthening
Color Key
−10
5
Value
TEL2
CDC28
RAD50
TOP3
CDC13
RFA1
TGS1
RAP1
TEN1
STN1
EST3
RIF2
SBA1
PIF1
EST2
OGG1
RFA2
EST1
HSP82
EBS1
RRM3
HEK2
TBF1
LCD1
Gene
Z_Shift_K_Doxo_HLD
Z_lm_K_Doxo_HLD
Z_Shift_K_Doxo_EG
Z_lm_K_Doxo_EG
Z_Shift_L_Doxo_HLD
Z_lm_L_Doxo_HLD
Z_Shift_L_Doxo_EG
Z_lm_L_Doxo_EG

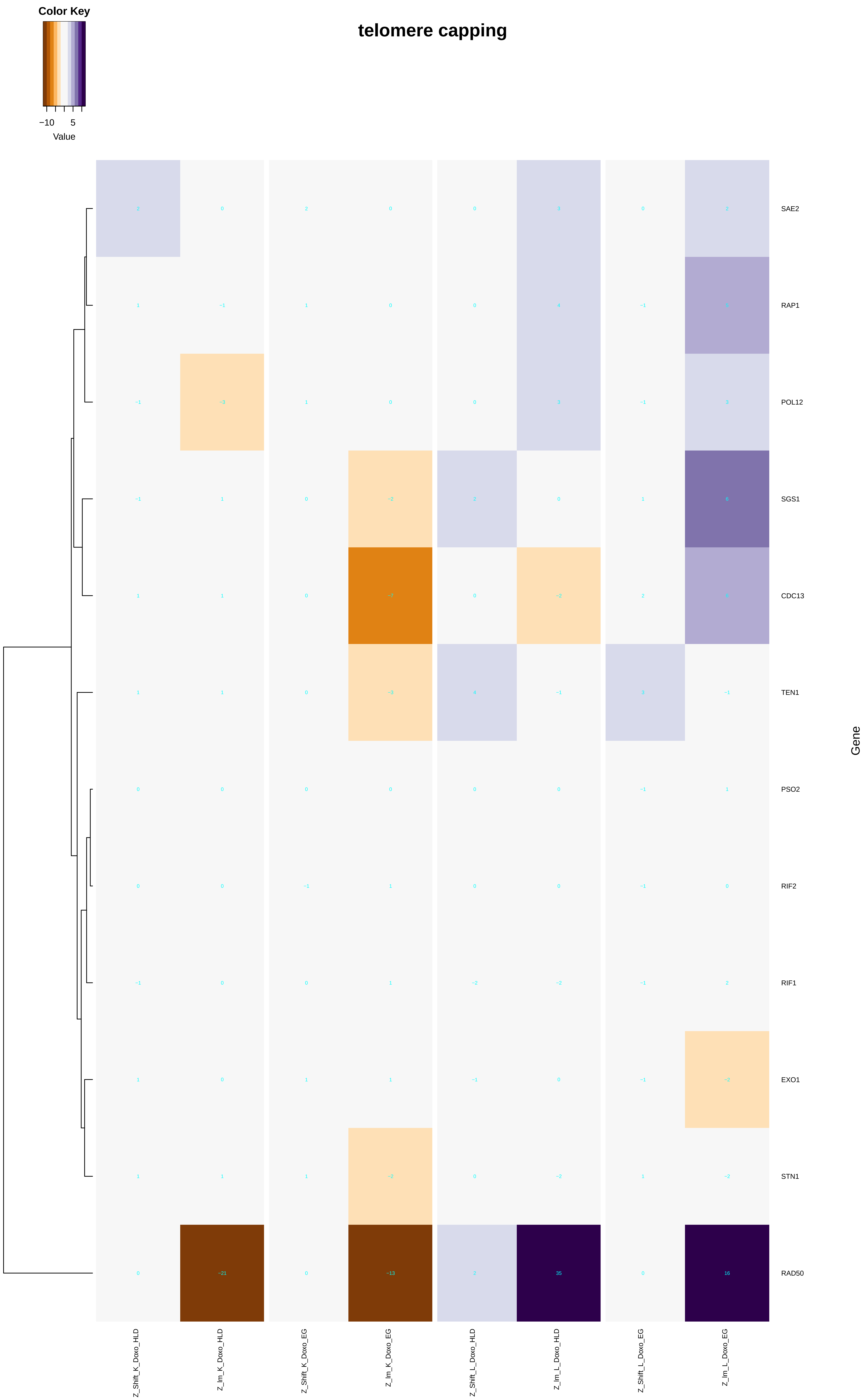

Color Key
−10
5
Value
telomere capping
SAE2
RAP1
POL12
SGS1
CDC13
TEN1
PSO2
RIF2
RIF1
EXO1
STN1
RAD50
Gene
Z_Shift_K_Doxo_HLD
Z_Im_K_Doxo_HLD
Z_Shift_K_Doxo_EG
Z_Im_K_Doxo_EG
Z_Shift_L_Doxo_HLD
Z_Im_L_Doxo_HLD
Z_Shift_L_Doxo_EG
Z_Im_L_Doxo_EG

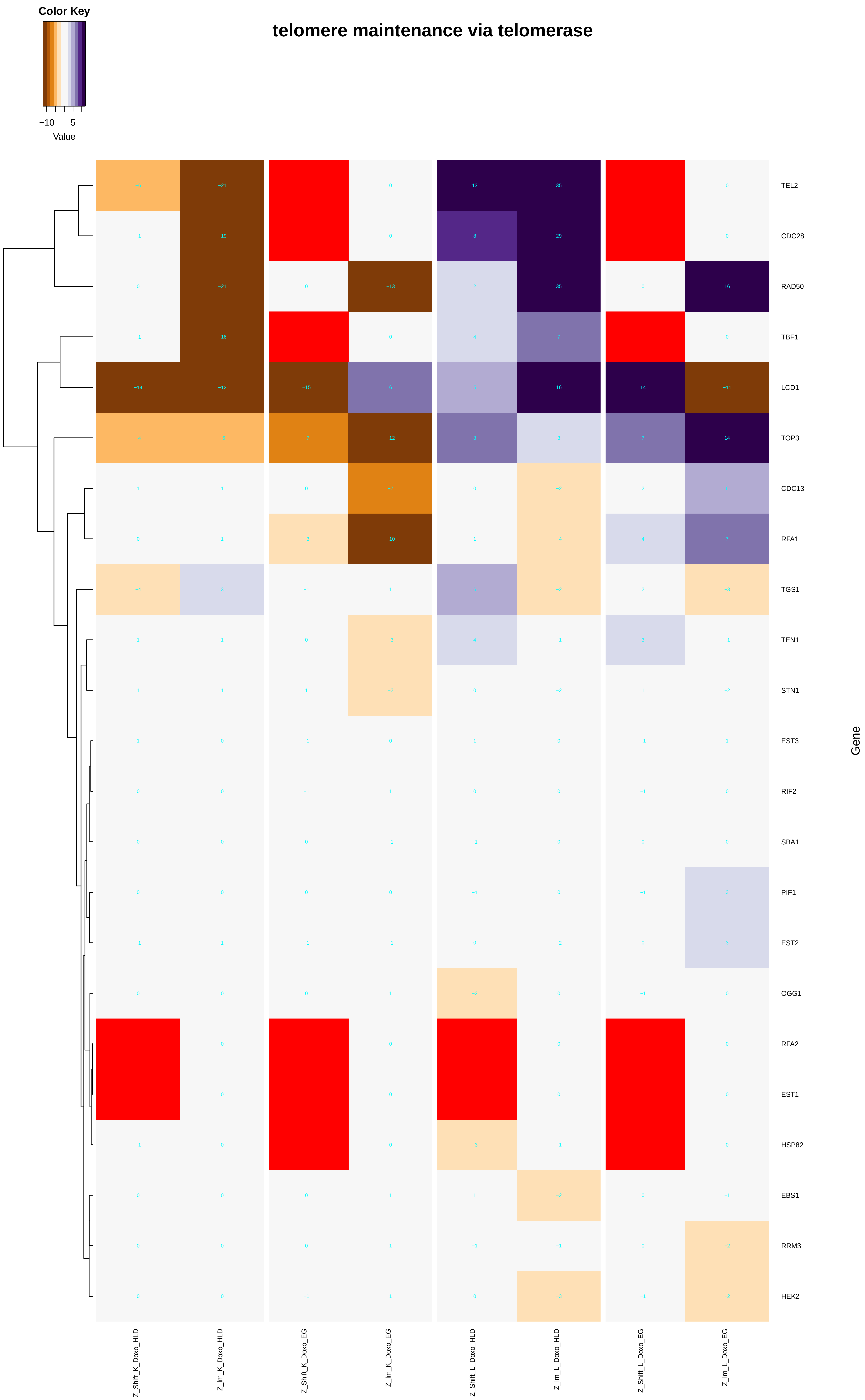

Color Key
−10
5
Value
telomere maintenance via telomerase
TEL2
CDC28
RAD50
TBF1
LCD1
TOP3
CDC13
RFA1
TGS1
TEN1
STN1
EST3
RIF2
SBA1
PIF1
EST2
OGG1
RFA2
EST1
HSP82
EBS1
RRM3
HEK2
Gene
Z_Shift_K_Doxo_HLD
Z_Im_K_Doxo_HLD
Z_Shift_K_Doxo_EG
Z_Im_K_Doxo_EG
Z_Shift_L_Doxo_HLD
Z_Im_L_Doxo_HLD
Z_Shift_L_Doxo_EG
Z_Im_L_Doxo_EG
